# Identification and Expression Pattern of EZH2 in Pig Developing Fetuses

**DOI:** 10.1155/2020/5315930

**Published:** 2020-10-05

**Authors:** Baohua Tan, Linjun Hong, Jiaxin Qiao, Jian Zhou, Pingping Xing, Guanhao Yan, Enqin Zheng, Gengyuan Cai, Sixiu Huang, Zhenfang Wu, Ting Gu

**Affiliations:** ^1^National Engineering Research Center for Breeding Swine Industry, Guangdong Provincial Key Lab of Agro-Animal Genomics and Molecular Breeding, College of Animal Science, South China Agricultural University, Guangzhou 510642, China; ^2^Wens Foodstuff Group Co., Ltd., Yunfu, China

## Abstract

The proper methylation status of histones is essential for appropriate cell lineage and organogenesis. EZH2, a methyltransferase catalyzing H3K27me3, has been abundantly studied in human and mouse embryonic development. The pig is an increasing important animal model for molecular study and pharmaceutical research. However, the transcript variant and temporal expression pattern of *EZH2* in the middle and late porcine fetus are still unknown. Here, we identified the coding sequence of the *EZH2* gene and characterized its expression pattern in fetal tissues of Duroc pigs at 65- and 90-day postcoitus (dpc). Our results showed that the coding sequence of *EZH2* was 2241 bp, encoding 746 amino acids. There were 9 amino acid insertions and an amino acid substitution in this transcript compared with the validated reference sequence in NCBI. *EZH2* was ubiquitously expressed in the fetal tissues of two time points with different expression levels. These results validated a different transcript in pigs and characterized its expression profile in fetal tissues of different gestation stages, which indicated that EZH2 played important roles during porcine embryonic development.

## 1. Introduction

Polycomb Group (PcG) proteins are a family of protein complex that regulate gene expression, especially repressing gene transcription [1]. As one of the two distinct complexes, namely, Polycomb Repressive Complex 1 (PRC1) and PRC2, PRC2 mediates gene silencing by modulating chromatin structure [2]. Enhancer of zeste homolog 2 (EZH2), one of the core components of PRC2, is a methyltransferase possessing the enzymatic activity to generate di/trimethylated lysine 27 in histone H3 [2]. Since identification of EZH2 in the research of protooncogene product Vav [3], studies have shown that EZH2 is highly expressed in tumorigenesis, which regulates the expression of tumor suppressor genes, such as in breast cancer, prostate cancer, and lung cancer [4–6]. In the early embryonic development, abnormal expression of EZH2 impaired embryo growth and pluripotency maintenance [7, 8]. Furthermore, the decreased expression of the *EZH2* gene is crucial for stem cell differentiation into specific cell lineages involved in myogenesis, adipogenesis, osteogenesis, neurogenesis, and haematopoiesis [9]. In cardiac conditional Ezh2-knockout mice, cardiomyocyte proliferation was repressed, and the apoptosis process was induced [10]. Thus, the appropriate expression level of the *EZH2* gene is important for embryonic development. So far, the expression pattern and the function of *EZH2* have been broadly studied in humans [11] and mice [12, 13]. In pigs, several studies have focused on its function in early embryonic development regulation and in SCNT efficiency improvement [8, 14–16]. However, the transcript and expression status of the *EZH2* gene during porcine embryonic development remains unknown.

Alternative splicing of gene can generate multiple transcripts and proteins to regulate tissue and organ development [17]. To date, a lot of *EZH2* variants have been found in various cell and tissue types [18–20]. Considering the varieties of *EZH2* splicing variant, identifying its transcript in porcine fetal tissues is the foundation to study its function. However, only one transcript has been validated in pigs, with several other transcript variants of *EZH2* from digital computational analysis in NCBI that have not been empirically validated. Moreover, none of the *EZH2* transcript variants have yet been identified in porcine fetal tissues.

In this study, we identified a transcript variant of *EZH2* in porcine fetal tissues by cloning and sequencing. Subsequently, we detected the expression of *EZH2* on mRNA level and protein level in two different embryonic development stages (65-dpc and 90-dpc) via qRT-PCR and western blots. The subcellular localization of EZH2 protein was predicted by using different predictors (CELLO, Euk-mPLoc, WoLF PSORT, and TargetP). Our study is aimed at providing information for understanding the biological function of EZH2 in porcine embryonic development.

## 2. Materials and Methods

### 2.1. Ethics Statement

All experiments were carried out following the rules of the Administration of Affairs Concerning Experimental Animals (Ministry of Science and Technology, China, revised June 2004) [21] and approved by the Animal Care and Use Committee of the South China Agricultural University, Guangzhou, China (approval number # SYXK2014-0136) [22].

### 2.2. Sample Collection

Six Duroc pig fetuses were used in our study, consisting of 3 fetuses on gestation day 65 and 3 fetuses on gestation day 90. In order to reduce the influence of individual differences on the results, each 3 fetuses on the same gestation day were half sibs. The definition of age was based on the time of fertilization. Each visible tissue including heart, liver, spleen, lung, kidney, and longissimus dorsi muscle was quickly sectioned in a 2 ml centrifuge tube and then stored in a liquid nitrogen tank until laboratory examinations.

### 2.3. Cloning and Sequencing

To acquire the coding sequence in fetal Duroc pigs, we extracted total RNA from the heart, liver, spleen, lung, kidney, and longissimus dorsi muscle on gestation 65 according to the instruction of Total RNA Kit I (Omega Bio-Tek, Norcross, USA) and then quantified it with spectrophotometry at 260/280 nm in a NanoDrop 2000 instrument (Thermo Fisher Scientific, Waltham, USA). By using a reverse transcription kit (TAKARA, Dalian, China), we synthesized cDNA in a PCR instrument (Life Technologies, NY, USA). The cDNA products were amplified by Taq DNA polymerase (Genstar, Beijing, China) with specific primer (forward primer: 5-ATGGGCCAGACTGGGAAGAA-3; reverse primer: 5-TCAAGGGATCTCCATTTCTCTTTCGAA-3). The primers were designed and produced by Sangon Biotech Co., Ltd. (Shanghai, China) with reference sequence in NCBI (accession no: NM_001244309.1). The PCR amplification steps were as follows: 94°C, 5 min for incubation; 94°C, 30 s, 57.3°C, 30 s; 72°C, 40 s, repeated 35 cycles; and 72°C, 10 min. The PCR product was separated using 2% agarose gel and purified with a Gel Extraction Kit (Omega Bio-Tek, Norcross, GA, USA). The purified products were ligated into the pMD19T simple vector (TAKARA, Dalian, China), and then, recombinant plasmids were transformed into *Escherichia coli* DH5*α* competent cells (TAKARA, Dalian, China). Transformed DH5*α* competent cells were smeared onto Luria-Bertani- (LB-) ampicillin plate medium and then incubated at 37°C for 12 h in a constant-temperature incubator. The target colonies were selected and mixed with 200 *μ*l LB-ampicillin medium. The mixtures were used as a cDNA template to amplify with specific primers as described above, and then, the existence of target gene was confirmed through electrophoresis. Finally, 8 identified colonies containing the target fragment of each tissue were sent to BGI (BGI, Shenzhen, China) for sequencing.

### 2.4. Bioinformatics Analysis

The cDNA sequence of *EZH2* derived from fetal tissue was deduced via the ProtParam software (https://web.expasy.org/translate/). Deduced protein sequence was aligned with the reference sequence (accession no: NP_ 001231238.1) via the DNAMAN software (Lynnon Biosoft, USA). Prediction of conserved domain was performed in NCBI (https://www.ncbi.nlm.nih.gov/Structure/cdd/wrpsb.cgi) [23]. The secondary structure was calculated by SOPMA (https://npsa-prabi.ibcp.fr/cgi-bin/npsa_automat.pl?page=npsa_sopma.html) [24]. Phyre 2.0 (http://www.sbg.bio.ic.ac.uk/phyre2/html/page.cgi?id=index) [25] and Pymol 2.0 (http://www.pymol.org) were used to predict tertiary structure. The subcellular localization of the EZH2 protein was predicted in Euk-mPLoc (http://www.csbio.sjtu.edu.cn/bioinf/euk-multi-2/) [26], CELLO (http://cello.life.nctu.edu.tw/) [27], WoLF PSORT (https://wolfpsort.hgc.jp/) [28], and TargetP (http://www.cbs.dtu.dk/services/TargetP-1.1/index.php) [29].

### 2.5. Quantitative Real-Time PCR Analysis

We had performed quantitative real-time PCR (qRT-PCR) to examine the expression pattern of *EZH2* mRNA in different tissues including liver, spleen, lung, kidney, and longissimus dorsi muscle on gestation days 65 and 90. To enable statistical analysis, three fully independent biological replicates and three technical repeats were conducted. The cDNA of each tissue from different individuals was synthesized as described above. Each reaction mixture (10 *μ*l) contained 1 *μ*l cDNA solution, 0.4 *μ*l of each specific primer, 5 *μ*l SYBR Select Master Mix (Thermo Fisher Scientific, Waltham, USA), and 3.4 *μ*l ddH_2_O. The primers were as follows: forward primer 5-CACGGCAGCCTTGCGACAG-3; reverse primer 5-CGGGAAAGCGGTTCTGACACTC-3. The reaction was performed in Quant Studio™ 7 Flex Real-Time PCR System (Thermo Fisher Scientific, Waltham, USA). The specificity of the PCR was confirmed through a single peak in the melting curve. Reaction conditions were as follows: 2 min at 50°C, 10 min at 95°C, then 45 cycles of 15 s at 95°C, 10 s at 60°C, and 15 s at 72°C, followed by melting curve analysis from 60°C to 95°C to evaluate the specificity of the PCR products. Relative expression in a given sample was calculated by normalizing to GAPDH mRNA level.

### 2.6. Western Blot Analysis

Western blot experiment was manipulated following the previous study [30]. Briefly, the total protein of tissues from different fetuses was extracted according to the direction of RIPA lysis buffer (Beyotime, Beijing, China). The concentration of protein was measured by a BCA protein essay kit (Thermo Fisher Scientific, Waltham, USA) and then adjusted to 30 *μ*g each hole for SDS-PAGE running. In this process, the proteins were subjected to immunoblotting analysis with the following antibodies: anti-EZH2 (1 : 1000, 5246S, Cell Signaling Technology, Danvers, Massachusetts, USA), anti-beta-tubulin (1 : 2000, GB11017, Servicebio, Wuhan, China), and HRP-conjugated Affinipure Goat Anti-Rabbit IgG (H+L) (1 : 5000, SA00001-2, Proteintech, Rosemont, IL, USA). Finally, the membranes were developed with the ECL system (WBULS0500, Millipore Corporation, Billerica, USA).

### 2.7. Statistical Analysis

The relative expression of EZH2 in the mRNA level and the protein level was visually shown in the figures presenting as mean ± standard error of the mean (SEM) from three independent individuals of each group. Quantification of transcript levels of the *EZH*2 gene was calculated by the comparative method (2^−*ΔΔ*Ct^) [31]. The band density value of the EZH2 protein was analyzed via ImageJ software [32]. Statistical analyses were performed by independent-sample Student's *t*-test in SPSS 20.0 software (IBM, Armonk, NY, USA). *P* < 0.05 indicates significant difference, and *P* < 0.01 indicates that the difference was extremely significant.

## 3. Results

### 3.1. Cloning and Sequence Analysis of Porcine EZH2 Gene

**Figure 1:**
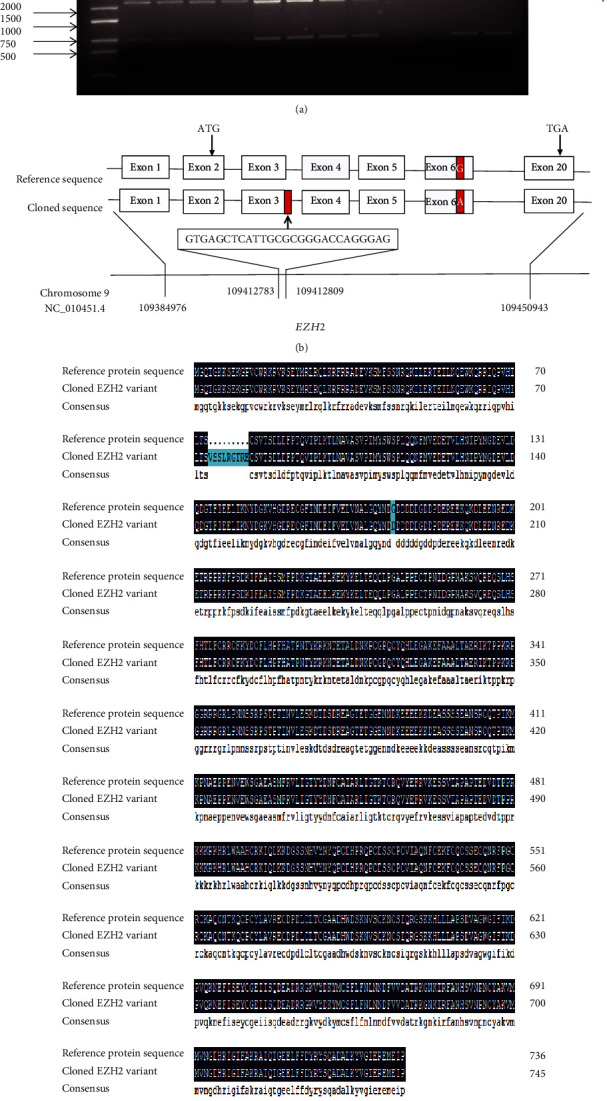
Sequence and molecular characteristics of cloned variant *EZH2*. (a) PCR amplification result of *EZH2* cDNA. M: DL5000 marker; lanes 1-2: heart; lanes 3-4: liver; lanes 5-6: spleen, lanes 7-8: lung; lanes 9-10: kidney; lanes 11-12: muscle. (b) Schematic representation of the genomic and alternative splicing of pig *EZH2* gene. The red boxes represent different bases. (c) Comparison of EZH2 protein amino acid sequence.

The PCR amplification product of *EZH2* was detected by 2% agarose gel electrophoresis. As expected, a specific band of approximately 2200 bp is shown in Figure 1(a). Sequencing analyses showed that cloned cDNA sequence of *EZH2* is 2241 bp and encoding 746 amino acids (accession no: MN_923188.1). The nucleotide sequences of *EZH2* were aligned with the validated reference sequences in NCBI. The alignment results showed that there was a 27 bp insertion and G→A mutation in the cloned coding sequence (Figure 1(b)). This 27 bp insertion was located at the position of g.109412783 to g.109412809 in genomic sequence of the *EZH2* gene (accession no: NC_010451.4). As shown in Figure 1(c), a 9 amino acid insertion and an amino acid substitution appeared in the cloned transcript of porcine fetal tissues.

### 3.2. Spatial Structures and Conserved Domain Analysis of EZH2 Protein

**Figure 2:**
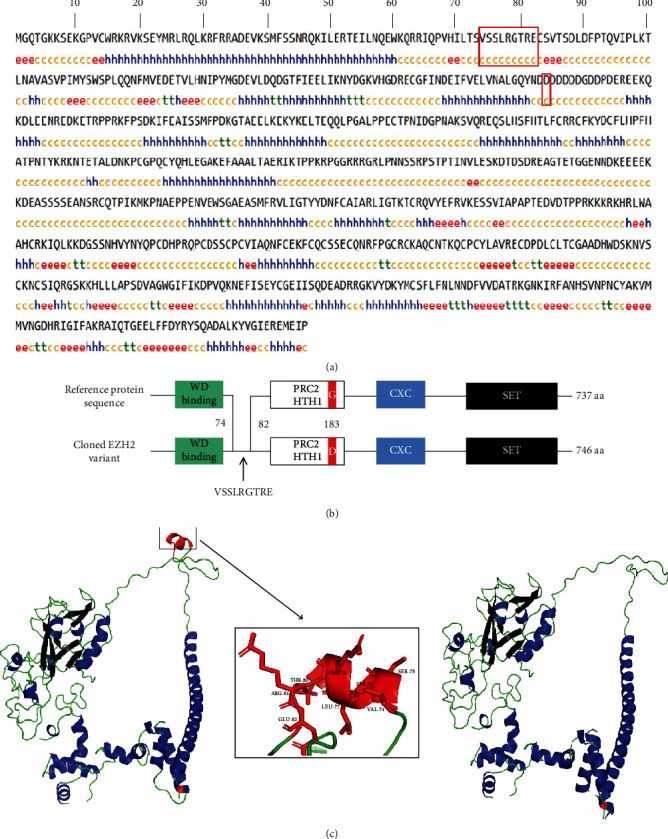
Spatial structures and domain analysis of EZH2 protein. (a) Secondary structure. The red boxes represent secondary structures of a 9 amino acid insertion and an amino acid substitution. h: alpha helix; e: extended strand; c: random coil; t: beta turn. (b) Comparison of conserved domain. The numbers represent the position of amino acid insertion and substitution. (c) Comparison of tertiary structure. Left: cloned sequence; middle: amino acid insertion; right: the reference sequence. Blue: alpha helix; black: beta sheet; green: the other structures except alpha helix and beta sheet; red: amino acid insertion and substitution.

The secondary structure analysis indicated that the EZH2 protein consisted of alpha helix (31.77%), extended strand (12.47%), beta turn (4.69%), and random coil (51.07%) (Figure 2(a)). For the newly cloned *EZH2* variant, a 9 amino acid insertion was composed of random coils. The amino acid substitution was composed of random coil and consistent with reference transcript. Additionally, the 9 amino acid insertion was not located in the conserved domain including WD-binding domain, CXC, SET, and Polycomb Repressive Complex 2 Tri-helical domain (PRC2 HTH 1), while the amino acid substitution was located in the PRC2 HTH 1 domain (Figure 2(b)). As shown in Figure 2(c), the tertiary structure result showed the protein encoded by our cloned transcript was mainly composed of alpha helix, beta sheet, and random loop. In particular, the amino acid insertion was mainly composed of alpha helixes, while the amino acid substitution did not change tertiary structure.

### 3.3. Subcellular Localization of EZH2 Protein

The deduced protein sequence was applied to predict subcellular localization of EZH2 protein via four well-developed predictors (Euk-mPLoc, CELLO, WoLF PSORT, and TargetP). As shown in Table 1, the TargetP result indicated that no secretory pathway signal peptide and mitochondrial targeting peptide were detected in the EZH2 protein. Euk-mPLoc, CELLO, and WoLF PSORT indicated that the EZH2 protein had the greatest probability to locate in the nucleus. In conclusion, these predicted results showed that the EZH2 protein might be located in the nucleus.

### 3.4. The Expression Pattern of *EZH2* mRNA in Tissues

**Figure 3:**
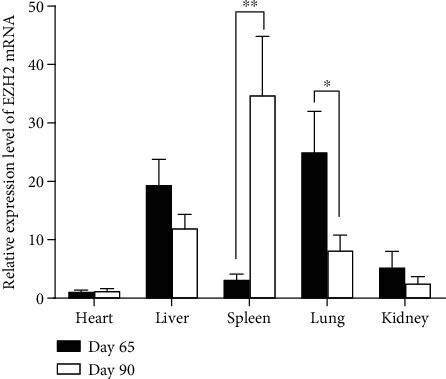
The qRT-PCR results of the *EZH2* gene in fetal tissues. The data shown in the figure are means ± SEM from three half sibs with the same gestation day. ^∗^*P* < 0.05 and ^∗∗^*P* < 0.01.

qRT-PCR was employed to detect the expression pattern of *EZH2* in the mRNA level during porcine embryonic development. As shown in Figure 3, *EZH2* was expressed ubiquitously in all tested tissues, including heart, liver, spleen, lung, kidney, and muscle. Statistical calculation demonstrated that *EZH2* was expressed with a significant difference in the spleen and lung. In the spleen, the mRNA level of *EZH2* significantly increased with the embryonic development (*P* < 0.01) while the opposite tendency of expression level was detected in the lung (*P* < 0.05). No significant difference of *EZH2* appeared in the heart, liver, kidney, and muscle between 65-dpc and 90-dpc. The result of muscle is shown in Supplementary Figure 1(a).

### 3.5. The Expression Pattern of EZH2 Protein in Tissues

**Figure 4:**
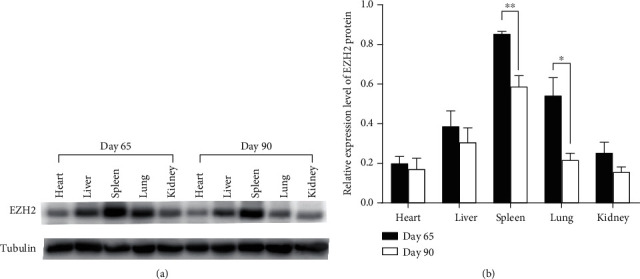
The western blot results of EZH2 in fetal tissues. (a) Western blot analysis for EZH2 protein. (b) Relative expression level of EZH2 protein. The data in the figure show means ± SEM from three half sibs with the same gestation day. ^∗^*P* < 0.05 and ^∗∗^*P* < 0.01.

The levels of the EZH2 protein were detected by western blot in various tissues, including heart, liver, spleen, lung, and kidney of Duroc pigs in 65-dpc and 90-dpc (Figure 4). Data indicated that the EZH2 protein was ubiquitously detectable in all tissues of two stages. Consistent with the result of qRT-PCR, there were no significant differences of EZH2 protein level in the heart, liver, muscle, and kidney. The expression of EZH2 showed a significant decrease in the spleen and lung from 65-dpc to 90-dpc (*P* < 0.01 and *P* < 0.05, respectively). The result of muscle is shown in Supplementary Figure 1(b).

## 4. Discussion

Genome-wide studies estimated that in 90–95% of human genes exist different levels of alternative splicing, which can generate various mRNA isoforms with different functions [17]. In eukaryotes, alternative splicing usually follows the GT-AG rule by both *cis*-elements and *trans*-acting factor regulation [33]. One of the most common types of alternative pre-mRNA splicing is alternative 5′ splice site, which is recognized by base paring with the end of the U1 small nuclear RNA (snRNA) [34]. Previous studies reported that alternative splicing variants of *EZH2* can influence biological effects of PRC2 to function in cell differentiation [18], central nervous system [19], and tumorigenesis [35]. In this study, we identified a transcript variant with a 27 bp insertion and a missense mutation from several porcine fetal tissues. For our cloned sequence, we found that the 27 bp insertion was located at the position of g.109412783 to g.109412809 in the genomic sequence of the EZH2 gene, which has been spliced out as part of intron 4 in the reference sequence previously reported. This indicated that it was not caused by DNA variation. Further, the bases of insertion followed the GT-AG rule and were located at the 5′ end of introns. Therefore, we drew a primary assumption that the transcript variant might be caused by the alternative 5′ splice site. By searching in the NCBI conserved domain database, we found that the insertion was present outside of the conserved domain. The missense G→A mutation occurred in the PRC2 HTH 1 domain which participates in the regulation of H3K27me3 as a part of the N-lobe of PRC2 [36]. This discrepancy with the reference sequence was also found in all other transcript variants of the *EZH2* gene in NCBI. We proposed that it may be caused by the inaccuracy of sequencing in the reference sequence. The specific reason needed further investigation. In terms of tertiary structure, we found that the alpha helix proportion was high in the EZH2 protein. Furthermore, amino acid insertion caused additional alpha helixes, which might increase the stability of the protein [37]. These results were useful for further study in the alternative splicing, conserved domain, and spatial structure of the EZH2 protein in porcine embryo.

Determining subcellular localization for a protein is significant to investigate its interaction partners, functions, and potential roles in different cells [38, 39]. In recent decades, many predictors varying on categories of sequence representation methods and classifiers have been proposed to predict protein subcellular localization with higher accuracy [28, 29, 40–43]. For example, WoLF PSORT is based on sorting signals, amino acid composition, and functional motifs to convert protein sequences into numerical localization features, and then, simple *k*-nearest neighbor classifier was used for prediction [28]. Wang et al. used WoLF PSORT to predict subcellular localization of Bv1 fxre and Bv6 nyuw and subsequently validated the prediction by lab experiments [44]. A similar validation was reported in VP1 protein [45]. Therefore, computational analysis can be applied to predict the subcellular localization of protein. A previous study demonstrated that *EZH2* is highly conserved in many species with about 90% homology [8]. The EZH2 protein was located in the nucleus in mouse embryonic cell and epithelial cells [46, 47] and human renal cell [48, 49]. In this study, the subcellular localization of the EZH2 protein in pigs was predicted by four well-developed predictors (Euk-mPLoc, CELLO, WoLF PSORT, and TargetP), rather than depending on a single predictor. Computational results predicted consistently that the EZH2 protein was located in the nucleus. Based on these findings, we speculated that EZH2 might exert function in the cell nucleus.

Understanding the spatial-temporal expression patterns of a gene is helpful to investigate its functions during the development of an organism [50]. Thus, we used qRT-PCR to detect the expression patterns of *EZH2* in different developmental stages of porcine embryo. Results showed that *EZH2* was ubiquitously expressed in tissues. The transcript abundance for *EZH2* varied significantly in the spleen and lung between 65-dpc and 90-dpc. Furthermore, we performed western blots to examine whether the EZH2 protein shared a similar expression pattern with that at the transcript level. Results showed that the EZH2 protein was expressed in all tested tissues, and its expression level appeared to have a decreasing tendency especially in the spleen and lung with development, which was consistent with the results in murine embryo [13, 46]. But in humans, EZH2 was highly expressed in the late stage of human embryonic development than in the middle stage [11], indicating that EZH2 might exert its function with different mechanisms in species. In particular, compared with the EZH2 protein, the expression level of *EZH2* mRNA exhibited an opposite tendency in the spleen from 65-dpc to 90-dpc. This finding suggested dynamic posttranscript modification of EZH2 in fetal spleen, as reported in astrocytic tumor cell [51]. Overall, our data provide foundation and clue to investigate the function of EZH2 in embryonic development, and further studies are still needed.

## 5. Conclusions

Our study validated a transcript variant of *EZH2* by cloning and sequencing in the fetal tissues of Duroc pigs. The sequence was 2241 bp, encoding 746 amino acids, with a 9 amino acid insertion and an amino acid substitution. The insertion caused more alpha helixes and was located outside of the conserved domain. The subcellular localization of the EZH2 protein was predicted in the nucleus. Additionally, *EZH2* was ubiquitously expressed in different fetal tissues, and the expression levels varied in different tissues and gestation stages. In conclusion, we provide a comprehensive analysis of sequencing, molecular characterization, and expression pattern of EZH2, which is helpful for further research on regulatory mechanism and function in porcine embryonic development.

## Figures and Tables

**Table 1:** Subcellular localization of the EZH2 protein predicted by four predictors.

Predictor	Predicated localization	Selected parameters
Euk-mPLoc	Nucleus	Default
CELLO^a^	Nucleus (4.750), cytoplasmic (0.133), extracellular (0.052), mitochondrial (0.018), chloroplast (0.013), plasma membrane (0.012), peroxisomal (0.007), ER (0.004), cytoskeletal (0.004), Golgi (0.003), vacuole (0.003), lysosomal (0.002)	Eukaryotes
WoLF PSORT^b^	Nucleus (26.0), nucleus and cytoplasmic (15.5), cytoplasmic (3), mitochondria (2), plastid (1)	Animal
TargetP^c^	mTP (0.139), SP (0.035), other (0.886)	Nonplant No cutoff set

^a^Output results in SVM score. ^b^32 nearest neighbors are used for this prediction and 26 of them indicated that EZH2 was located in the nucleus. ^c^Reliability class from 1 to 5 was used to score the prediction accuracy, while 1 indicates the strongest prediction. mTP: mitochondrial targeting peptide; SP: signal peptide.

## Data Availability

All the data used to support the findings of this study are included within the article.

## Supplementary Materials

Supplementary Materials Supplementary Figure 1: EZH2 expression in muscle. (a) The expression of EZH2 mRNA in muscle. (b) The expression of EZH2 protein in muscle.
