# Supplementary figures and images for: Identification and Expression Pattern of EZH2 in Pig Developing Fetuses
Source: Biomed Res Int. 2020 Oct 5;2020:5315930. doi: 10.1155/2020/5315930 (PMC7557918; doi:10.1155/2020/5315930)

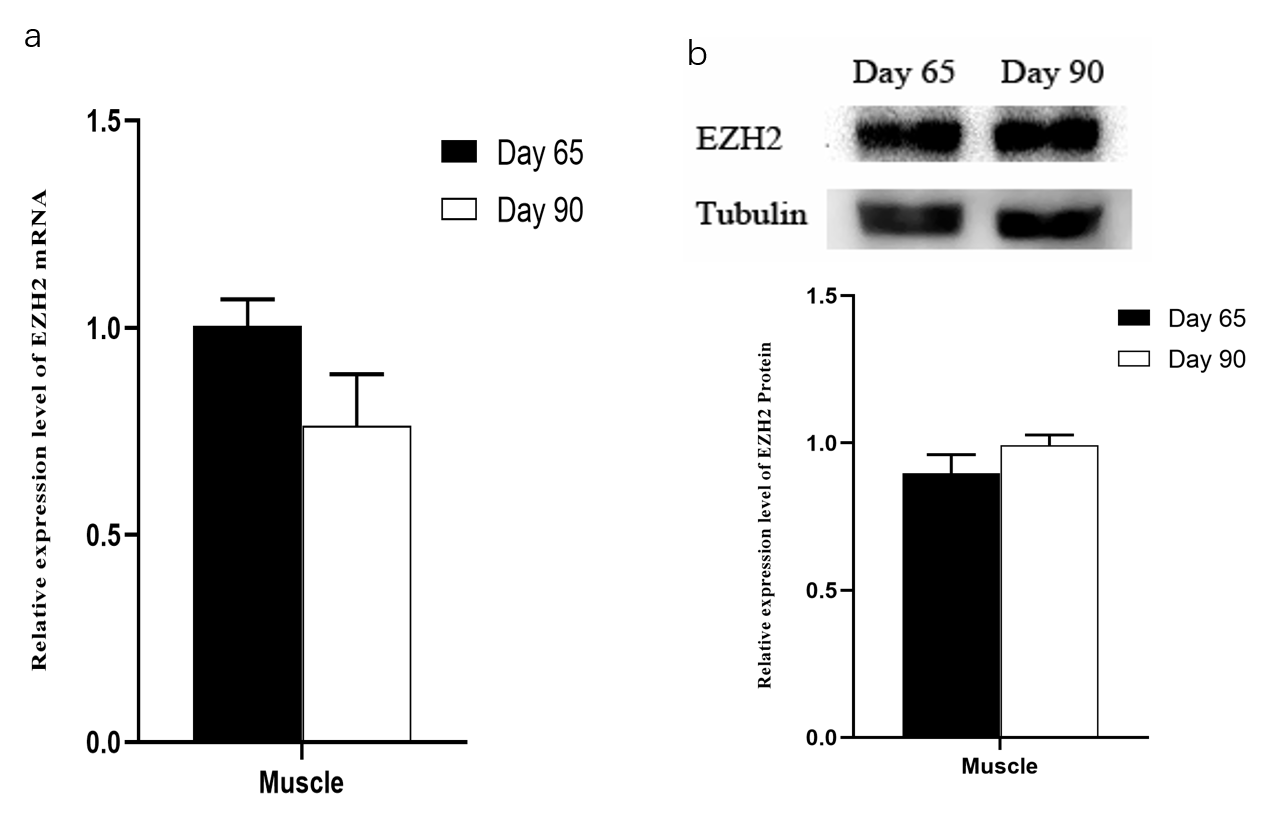

Supplement: Supplementary Materials — Supplementary Figure 1: EZH2 expression in muscle. (a) The expression of EZH2 mRNA in muscle. (b) The expression of EZH2 protein in muscle. [file 5315930.f1.docx]
